# The effect of dopaminergic neuron transplantation and melatonin co-administration on oxidative stress-induced cell death in Parkinson’s disease

**DOI:** 10.1007/s11011-022-01021-5

**Published:** 2022-09-08

**Authors:** Azam Asemi-Rad, Maral Moafi, Abbas Aliaghaei, Hojjat-Allah Abbaszadeh, Mohammad-Amin Abdollahifar, Mohammad-Javad Ebrahimi, Mohammad Hasan Heidari, Yousef Sadeghi

**Affiliations:** 1 Anatomy Department, School of Medicine, Zahedan University of Medical Sciences, Zahedan, Iran; 2 Anatomy and Biology Department, School of Medicine, Shahid Beheshti University of Medical Sciences, Tehran, Iran; 3 Hearing Disorders Research Center, Loghman Hakim Hospital, Shahid Beheshti University of Medical Sciences, Tehran, Iran; 4 Laser Application in Medical Sciences Research Center, Shahid Beheshti University of Medical Sciences, Tehran, Iran; 5 Department of Anatomy and Neuroscience, Faculty of Medicine, Dentistry and Health Sciences, University of Melbourne, Melbourne, VlC Australia

**Keywords:** Adipose Tissue-Derived Mesenchymal Stem Cell, Caspase 3, Melatonin, Oxidative stress, Parkinson’s disease, 6-Hydroxydopamine

## Abstract

A gradual degeneration of the striatum and loss of nigral dopamine cells are characteristic of Parkinson's disease. Nowadays, combination therapy for neurodegenerative disease is considered. This study aimed to investigate the effects of melatonin and dopaminergic neurons derived from adipose tissue stem cells (ADSCs) in a rat model of Parkinson’s disease. Parkinson’s disease was induced in rats using neurotoxin 6-Hydroxydopamine. The treatment was performed using melatonin and dopaminergic neurons transplantation. Subsequently, behavioral tests, western blot analysis for Caspase-3 expression, GSH (Glutathione) content and stereology analysis for the volume and cell number of substantia nigra and striatum were performed. Treatment with melatonin and dopaminergic neuron transplantation increased the number of neurons in substantia nigra and striatum while the number of glial cell and the volume of substantia nigra and striatum did not show significant change between groups. Western blot analysis for caspase 3 indicated the significant differences between groups. The results also indicated the increased level of glutathione (GSH) content in treatment groups. this study showed that combination therapy with melatonin and dopaminergic neurons could greatly protect the neurons, reduce oxidative stress and improve the symptoms of PD.

## Introduction

Parkinson’s disease (PD) is a progressive neurodegenerative disease and second most common neurodegenerative disorder, following Alzheimer’s disease (Dauer and Przedborski 2003; Miller and O’Callaghan 2015). In industrialized countries, the prevalence of PD is approximately 3% in the general population. The age of onset is about 60 years, although 5% to 10% of patients develop the disease at younger age (20–50 years) (Dexter and Jenner 2013). According to some studies, the prevalence is higher in males, while others have not reported any gender differences (Blauwendraat et al. 2021).

The main symptoms of PD include resting tremor, rigidity, and imbalance while walking. Bradykinesia is also a symptom of the disease, which is evident during activities, such as walking, standing, physical exercise, and dressing (Han et al. 2015; Noyce et al. 2012; Van Maele-Fabry et al. 2012). Although the pathology of PD depends on different factors, gradual degeneration of dopamine-producing neurons in the midbrain is responsible for the incidence of symptoms due to neuron loss in the substantia nigra pars compacta (SNpc) (Davie 2008; García-García et al. 2017; Kehagia et al. 2010). SNpc contains dopaminergic neurons whose terminals of dopaminergic neurons enter the striatum. Various studies have reported a 50–70% reduction in the number of dopaminergic neurons, which can produce PD symptoms. Therefore, one of the primary therapeutic approaches is based on dopaminergic neurons loss in SNpc or release of dopamine in the striatum The first choice in such a situation is either daily oral carbidopa/levodopa as dopamine replacement or a dopamine agonist such as apomorphine, Pramipexole, and Ropinirole which stimulate dopamine receptors extend the duration of the endogenous dopamine synthesis process. (Dauer and Przedborski 2003; Michel et al. 2016).

6-Hydroxydopamine (6-OHDA) is a hydroxylation analogue of dopamine, which was first isolated by Senob in 1959. The biological effects of 6-OHDA were first examined by Porter et al. (Senoh et al. 1959). This toxin can destroy neurons in the sympathetic system, while it cannot cross the blood–brain barrier; therefore, it should be prescribed intracranial. Several studies have shown that 6-OHDA is induced by a non-enzymatic process, spontaneous oxidation, and production of hydrogen peroxide, quinone, superoxide radicals, and hydroxyl radicals (Cannon and Greenamyre 2010; Tieu 2011).

Adipose tissue-derived stem cells (ADSCs) have a high proliferation capacity in laboratory conditions and differentiate into cells with characteristics of neural cells. Transplantation of ADSCs does not cause complications, such as tumor, chromosomal abnormalities, or rejection (Park et al. 2015). Some studies have shown that ADSCs, which are implicated in neuronal tissues, can produce host cell phenotypes. Stem cells like ADSCs have been used for cell therapy in numerous neurological diseases (Park et al. 2015).

Melatonin is a derivative of tryptophan, identified and isolated in 1958 by Lerner et al. (Iuvone et al. 2005). This hormone is synthesized by pinealocyte cells in the pineal gland, and its synthesis and release are regulated by seasonal changes throughout the day and night (Fleury et al. 2002). It is a potent antioxidant, which was first studied by Lanas and colleagues. Evidence suggests that this hormone exhibits antioxidant activity at low concentrations. Exogenous melatonin is used to treat various diseases, such as insomnia, stress, and neurodegenerative diseases.

Any increase in the amount of free radicals destroys lipids, proteins, and DNA, leading to necrosis or apoptosis of neurons. Jener et al. observed structural changes in lipids, proteins, and nucleic acids of nigrostriatal dopaminergic neurons induced by oxidative stress (Rios et al. 2010). In addition, Mayo et al. examined the ability of melatonin to prevent apoptosis in a model of PD and found that this hormone plays a role in the elimination of free radicals and inhibition of neoplastic apoptosis (Mayo et al. 1999).

Several studies have shown the role of melatonin as an effective element in the treatment of PD and other neurodegenerative diseases. Despite the use of cell therapy for PD and the importance of antioxidants in its treatment, use of these approaches has not been studied so far. Therefore, the aim of this study was to investigate the therapeutic effects of cell therapy with ADSC-derived dopaminergic neurons and melatonin co-administration on neuroprotection and movement disability associated with 6-OHDA-induced PD.

## Materials and methods

### Animals

A total of 96 adult male Sprague–Dawley rats (250 ± 20 g) were obtained from the laboratory animal center of Shahid Beheshti University of Medical Sciences, Tehran, Iran. The animal experiments were approved by the ethics committee of the university (IR.SBMU.MSP.REC.1395.243). The animals were kept under standard conditions (temperature 22–24 °C) in a 12:12 h light/dark cycle with humidity (50–70%) and free access to water and food.

### Experimental design

The animals were divided into eight groups randomly, each consisting of 12 animals: normal control with no surgical or injection procedure (group I); 6-OHDA (4 µg/kg, group II); 6-OHDA + cell transplantation (group III); 6-OHDA + cell transplantation + melatonin (group IV); 6-OHDA + melatonin (20 mg/kg; group V); vehicle (group VI); melatonin sham (group VII); and 6-OHDA sham (group VIII).

### Simulation of the experimental model of PD

The animals were anesthetized intraperitoneally with a mixture (1 ml/kg), containing 9 mg/kg of xylazine and 90 mg/kg of ketamine. Induction of PD in animal was performed by a unilateral single-dose injection of 6-OHDA (4 µg; Sigma, USA) in 2 µL of physiological saline (containing 0.02% ascorbic acid) into the right SN, using a Hamilton micro syringe (Hamilton, Reno, NV, USA) (Jeon et al. 1995). Coordinates were set according to the Atlas of Paxinos (Paxinos and Watson 2006): anteroposterior (AP): 4.3 mm; lateral (L): 1.6 mm; and dorsoventral (DV): 8.2 mm.

### Apomorphine-induced turning behavior

In order to ensure proper induction of PD, apomorphine-induced turning behavior was induced in all animals. Accordingly, the animals received a subcutaneous neck injection of apomorphine hydrochloride (0.05 mg/kg; Sigma, USA), in 1% ascorbic acid and 0.9% NaCl, and were placed on metal testing bowls for 30 min. Next, the number of contralateral rotations was recorded and analyzed.

### Cell transplantation

Differentiated cells were collected at approximately 2 × 10^4^ cell μL for transplantation. Cell transplantation was performed, using a unilateral stereotaxic injection with a Hamilton microsyringe. Coordinates were set according to the atlas of Paxinos (9): anteroposterior (AP): 9.2 mm; lateral (L): 3 mm; and dorsoventral (DV): 4.5 mm. Each animal received a slow infusion of cell suspension (2.5 μL) for 3–5 min.

### Melatonin treatment

Melatonin (sigma, USA) was dissolved in 2% DMSO and 98% Miglyol® 812 N to preparing 10 mg/mL concentration; it was protected from light and prepared fresh each time. The total administration dose was 20 mg/kg/day. It was initiated three days after 6-OHDA treatment and continued for seven days.

### Assessment of motor coordination

For evaluation of balance, coordination, and motor control of rats, the rotarod test was used, based on a linear accelerating protocol (4–40 rpm in 300 s). Three trials were carried out for each rat per day, and the average latency to fall was considered for each day. The test was performed one week after 6-OHDA injection, and continued for four consecutive weeks. The rats were sacrificed after the tests, and their brains were extracted for laboratory analyses.

### Tissue preparation

The rats were deeply anesthetized and sacrificed with sodium pentobarbital (60 mg/kg, Eutasil) intraperitoneally. Afterwards, trascardial perfusion was performed using normal saline containing 4% paraformaldehyde (PFA) in 0.1 M phosphate-buffered saline (PBS). All animal brains were exposed by a midline incision along the skull,next, they were dissected. Four brains from each group were immediately stored at -80 °C for molecular tests, while others were immediately immersed in a fresh 10% formalin solution for one week. Subsequently, the samples were embedded in paraffin blocks. In addition, complete serial Sects. (5–10 μm) were prepared using a microtome for stereological study. Every 10 sections were sampled. Next, 8–10 tissue sections were obtained from each animal in a systematic random sampling with a random number between1-10. Finally, the selected sections were stained with haematoxylin and eosin (H&E) and analyzed.

### Western blot assays

Following behavioral tests, Western blotting was carried out to investigate caspase-3 expression in the striatum and SNpc. The rats were sacrificed under ketamine/xylazine anesthesia after decapitating their brains (striatum and SNpc). Tissue samples were homogenized in tissue lysis buffer (1:10 w/v; Sigma). Next, protein concentrations were determined based on Bradford assay. Protein samples (30 lg) were separated by 10% sodium dodecyl sulfate polyacrylamide gel electrophoresis (SDS-PAGE) and transferred onto a nitrocellulose membrane (Amersham Biosciences, Piscataway, NJ, USA). After blocking the membrane in 10% milk with TBS-T buffer (10 mM Tris–HCl, 120 mM NaCl, and 0.1% Tween-20; pH: 7.4) for one hour at room temperature, it was incubated with caspase-3 primary antibodies (GeneTex Cat# GTX22300, RRID:AB_367945) (1:1000) at 4 °C overnight. It was then washed three times in TBST buffer, incubated with 1:10,000 dilutions of HRP-conjugated anti-rabbit/goat IgG at room temperature for one hour. Visualization was carried out using an Excellent Chemiluminescent Substrate (ECL) kit (GE Healthcare, Bucks, UK). Density of the bands on Western blots was also quantified through densitometric analysis of scanned blots in ImageQuant software.

### Measurement of GSH assay

Reduced glutathione (GSH) reacts with 5–5–dithio-bis-2(nitrobenzoic acid) (DTNB) to form a yellow dianion of 5′thio-2-nitrobenzoic acid (TNB) which is measured by its absorbance at 412 nm.

### Stereological analysis

#### Estimation of striatum and SN volume

The rats’ striatum and SN tissue samples were fixed in 4% PFA for one week. Following tissue processing, serial coronal Sects. (25 mm) were prepared and stained with Cresyl violet 0.1%. Cresyl violet was used to demonstrate the Nissl substance in neurons. For volume estimation, 10–12 sections per block were selected through systematic uniform random sampling. Stereological studies were carried out using a projection microscope. To measure the total volume of striatum and SN at 25 × magnification, the Cavalieri's Principle was used based on the following formula (Mayo et al. 1999):

$$\mathrm V(\mathrm{striatum}\;\mathrm{and}\;\mathrm{SN})=(\mathrm a/\mathrm p)\times\mathrm\Sigma\mathrm P(\mathrm{striatum}\;\mathrm{and}\;\mathrm{SN})\times\mathrm d$$

Moreover, distance between the sampled sections (d) was calculated. Also, the section area was estimated using the point-counting method. The area per point (a/p) was 0.36 mm^2^, and on average, 500 points were counted per animal.

#### Estimation of neuron and glial cell count

The total number of neurons and glial cells in the striatum was determined using the optical disector technique. Microscopic fields were selected at equal intervals of stage movement, based on systematic uniform random sampling (SURS). A microcator was used for measuring the Z-axis movement of the microscope stage. An unbiased counting frame with inclusion and exclusion borders was superimposed on the images, viewed on the monitor. The nucleus count was determined if the nuclei were placed completely or partially within the counting frame and did not reach the exclusion line. Numerical density (Nv) was also calculated based on the following formula:

$${\mathrm{N}}_{\mathrm{v}}=\frac{\sum Q}{\sum P\times h\times \frac{a}{f}} \times \frac{t}{BA}$$

where “ΣQ” represents the number of nuclei, “ΣP” denotes the total number of unbiased counting frames in all fields, “h” is the height of the dissector, “a/f” is the frame area, “t” is the real section thickness measured in every field by the microcator, and “BA” is the block advance of microtome set at 10 μm. The total number of neurons and glial cells was estimated by multiplying the numerical density (Nv) by the total volume.

$${\mathrm{N}}_{\mathrm{total}}={\mathrm{N}}_{\mathrm{v}}\times \mathrm{v}$$

#### Estimation of coefficient error(CE)

For volume estimations, CE was calculated using the following formula: P_i_.^2^

$$\frac{1}{240\{3\sum {p}_{i }^{2 }+ \sum {p}_{i }-4\sum {p}_{i }+0.0724+ \frac{B}{\sqrt{A}} \times \surd n\sum {p}_{i }\}{~}^{1}\!\left/ \!{~}_{2}\right.}$$

where “B” and “A” represent the mean section boundary length and mean section area, respectively. For estimating the total neuron and glial cell count, CE was derived from CE (V) and CE (Nv), based on the following formula.

(Gundersen and Jensen 1987):

$${\mathrm{CE}(\mathrm{N})=[{\mathrm{CE}}^{2}({\mathrm{N}}_{\mathrm{v}})+{\mathrm{CE}}^{2}(\mathrm{v})]}_{1/2}$$

$${\mathrm{CE}({\mathrm N}_{\mathrm v})=\lbrack(\frac n{n-1})\times\lbrack\left(\frac{\sum({Q)}^2}{(\sum{Q)}^2}\right)+\left(\frac{\sum({P)}^2}{(\sum{P)}^2}\right)-\left(\frac{2\sum\left(QP\right)}{\left(\sum Q\sum P\right)}\right)\rbrack\rbrack}\frac12$$

### Data analysis

Statistical analysis between the groups was examined using ANOVA and Tukey’s post hoc test. All statistical analysis was performed in SPSS version 24 (SPSS Inc., Chicago, USA), and P < 0.05 was considered significant.

## Results

### Apomorphine-induced turning behavior

**Fig. 1:**
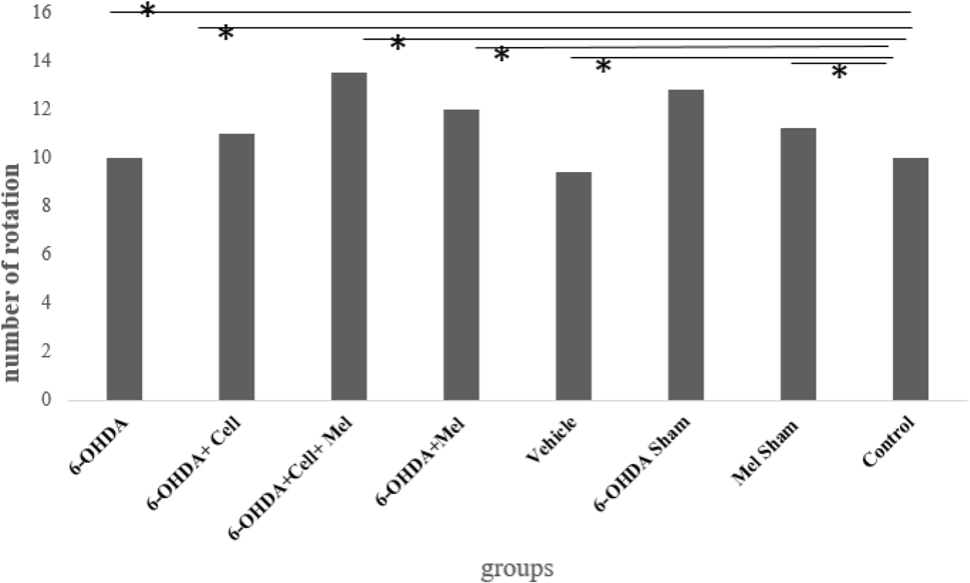
Number of rotations induced by apomorphine injection in 6-OHDA-injected, treatment groups of rats compared with healthy control group (*, *P* < 0.05; **P < 0.01, ***P < 0.001)

The results of apomorphine-induced rotation test showed that animals with 6-OHDA lesions were considered to have nearly complete lesions with more than 100 rotations in 30 min, whereas the control animals showed less than 10 rotations. Statistical analysis showed that the number of rotations in the 6-OHDA group was significantly higher in comparison with the control group (P < 0.0.5) (Fig. 1).

### Motor coordination

**Fig. 2:**
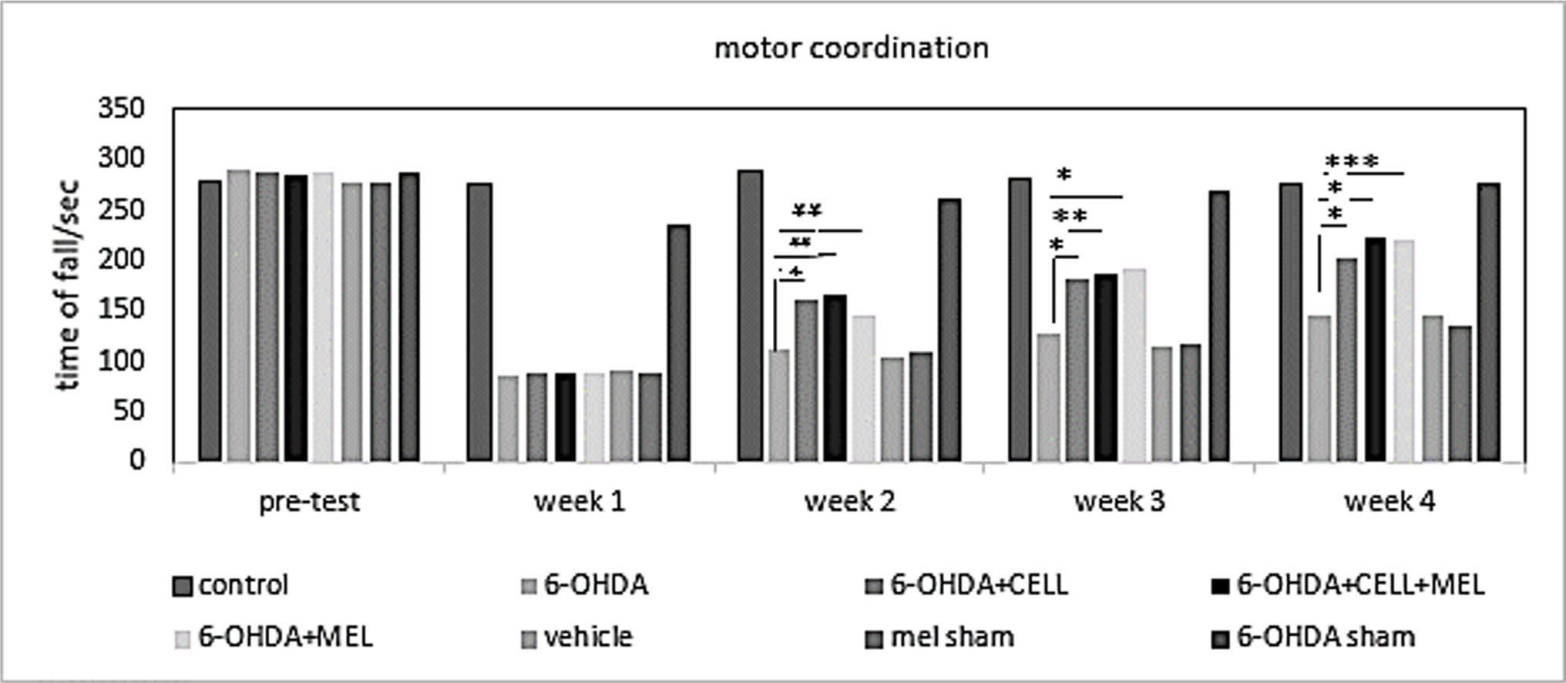
The administration of cell transplantation and melatonin in 6-OHDA-injected rats improved motor coordination. Number sign indicates the difference between the 6-OHDA-injected group and treatment group, cell transplant, melatonin and cell transplant + melatonin (*, *P* < 0.05; **, *P* < 0.01; ***, *P* < 0.001)

The rotarod motor coordination test was performed in the test groups before the induction of PD model and up to four weeks after induction. Before induction of the PD model, there was not any significant differences between the groups. In the first test, there was a significant difference between the healthy and 6-OHDA groups after the induction of PD model (P < 0.05). In the second test, which was performed at the end of the second week of model induction, the results showed that the healthy controls were significantly different from the 6-OHDA, cell transplantation, melatonin, and cell transplantation + melatonin groups (P < 0.05). Finally, in the third and fourth tests, the cell transplantation, melatonin, and cell transplantation + melatonin groups were significantly different from the healthy control and 6-OHDA groups (P < 0.05) (Fig. 2).

### Western blotting

**Fig.3:**
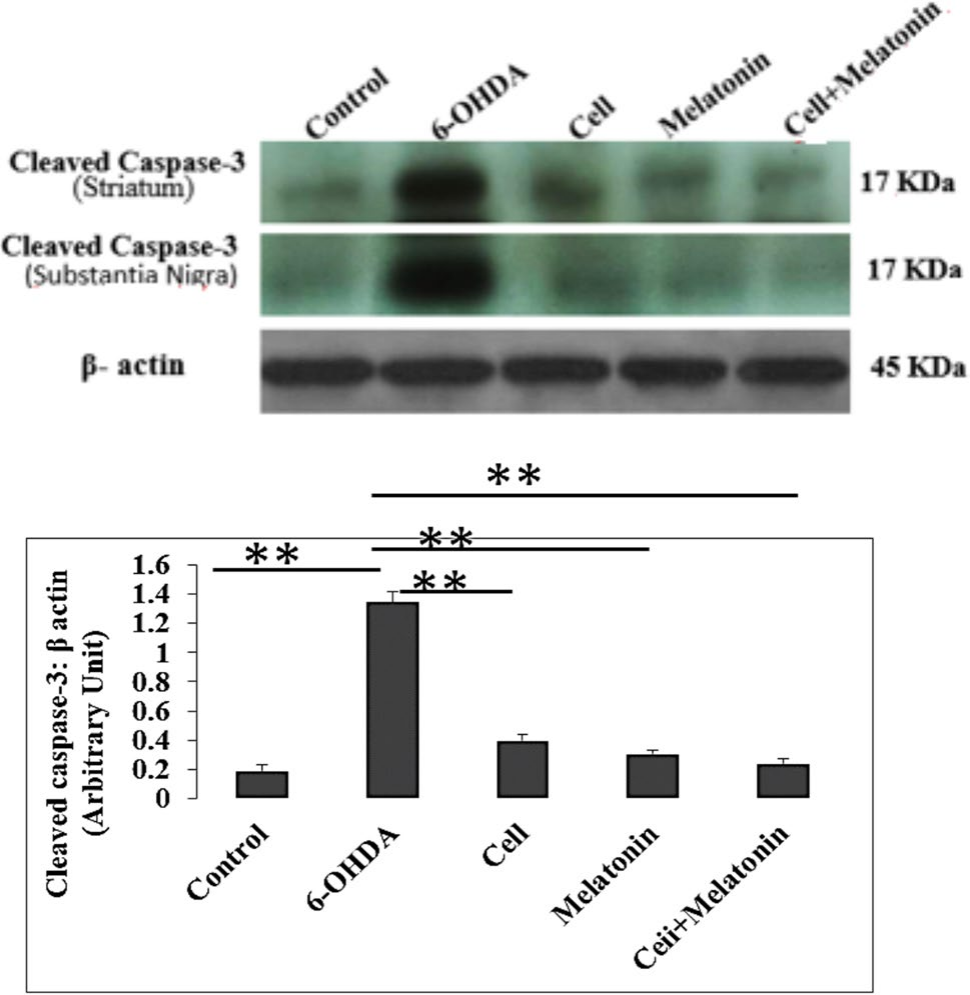
Western blot analysis to evaluate the effect of cell transplantation and melatonin treatment on the expression levels of an apoptotic marker Caspase-3 in 6-OHDA-injected rats. **A** Western blot for cleaved caspase-3 are shown. Equal amounts of total proteins were separated by SDS-PAGE and blots were probed with anti-caspase-3 and anti β-actin antibody. The results showed high expression of cleaved caspase-3 in 6-OHDA-injected rats. **B** The densities of cleaved caspase-3 bands were measured and the ratios to βactin bands were calculated. Number sign indicates the difference between the 6-OHDA-injected, treatment and control groups (*, *P* < 0.05; **, *P* < 0.01; ***, *P* < 0.001)

To evaluate the 6-OHDA ability to induce apoptosis, caspase-3 activity was examined. The results showed that caspase-3 activity significantly reduced in the treatment groups (cell transplantation, melatonin, and cell transplantation + melatonin groups), compared to the 6-OHDA group (P < 0.05) (Fig. 3).

### Antioxidant tests

**Fig.4:**
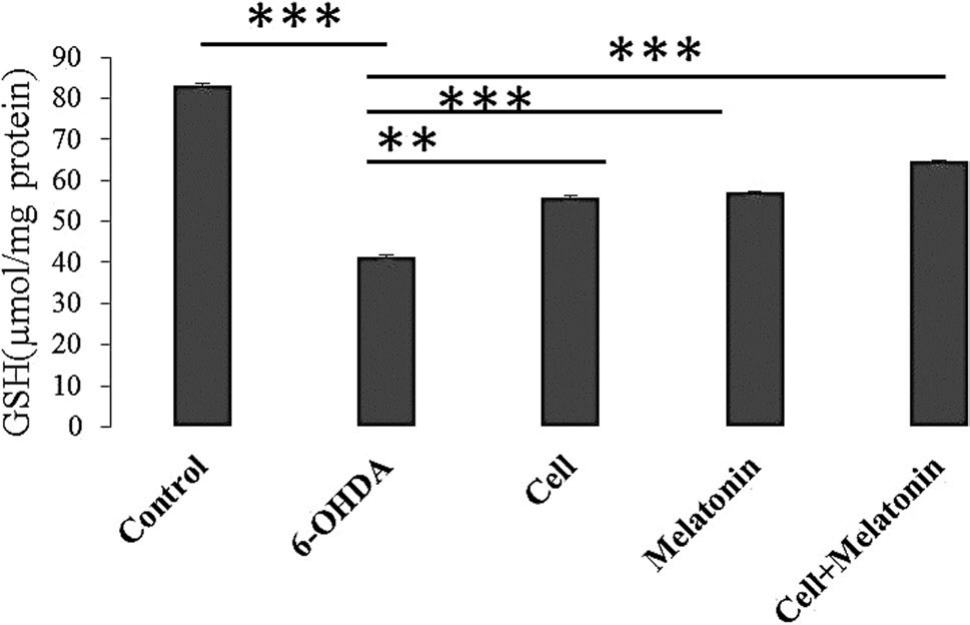
The effect of cell transplantation and melatonin treatment on changes in GSH content in rats receiving 6-OHDA. Each point shows the mean ± SEM. (*, *P* < 0.05; **, *P* < 0.01; ***, *P* < 0.001)

GSH activity was evaluated using the Ellman's assay. The results revealed that the expression of this enzyme was significantly lower in the 6-OHDA group, compared to the control group (P < 0.001). On the other hand, GSH activity increased significantly in the treatment groups, compared to the 6-OHDA group (P < 0.001) (Fig. 4).

### Stereology

**Fig. 5:**
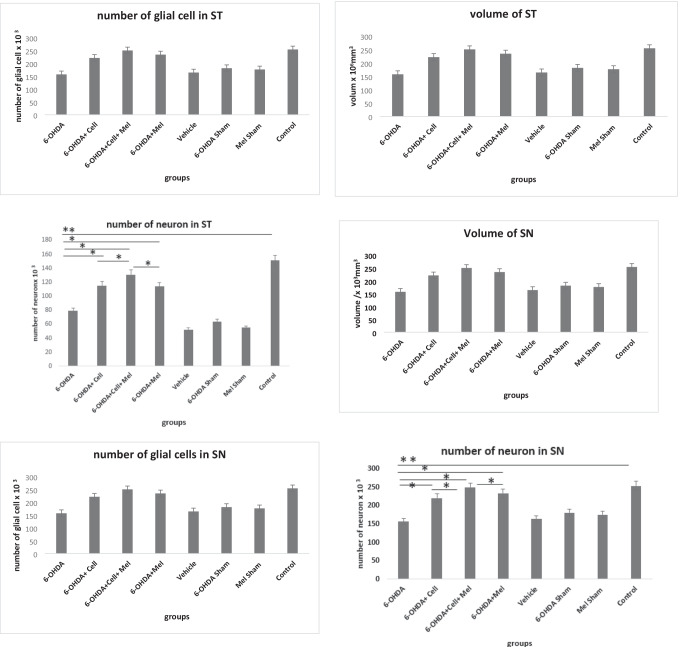
The volume and the number of neurons and glial cells in striatum (ST) and substantia Nigra (SN) in 6-OHDA-injected, treatment and control groups using stereology method. The volume and number of glial cell in striatum(ST) and Substantia Nigra (SN) was not significant. (*, *P* < 0.05; **, *P* < 0.01; ***, *P* < 0.001)

The number of neurons in the striatum and SN(pc) decreased significantly in the 6-OHDA group, compared to the healthy control group (P < 0.05). However, there was no significant change in the striatum and SN(pc) volume and glial cell count, compared to the healthy control group. Stereological results indicated that the number of neurons significantly increased after treatment with cell transplantation, melatonin, and cell transplantation + melatonin, compared to the 6-OHDA group (P < 0.05). Therefore, the striatum and SN(pc) volume and glial cell count did not significantly change in the treatment groups, compared to the 6-OHDA group (Fig. 5).

## Discussion

To develop an experimental model of PD, various neurotoxins are used in the SN and medial forebrain bundle. In the present study, 6-OHDA, which was selectively absorbed by dopaminergic neurons was injected in the SN to develop a PD model in rats. A dose of 4 μg/8 μL was selected based on previous studies, which resulted in the destruction of SN and unilateral mesencephalic lesions in the ventral tegmental area (Agrawal et al. 2004). Motor evaluation based on the rotarod test showed a significant difference between 6-OHDA and healthy control groups. The results also showed a significant improvement in the melatonin, cell transplantation, and melatonin + cell transplantation groups.

The present results are similar to those reported by Lauretti et al. on MPTP-induced PD rats. In their study, the rotarod test showed that MPTP-induced PD rats had motor deficits, unlike the healthy group (Lauretti et al. 2016). The results of rotarod test in the melatonin, cell transplantation, and melatonin + cell transplantation groups indicated a significant increase in travel time on rotarod rotational axis, compared to the 6-OHDA group. The results of the present study are similar to those reported by Kirik et al., which showed that motor deficits improved in 6-OHDA-induced PD rats after using I-DOPA (Kirik et al. 2002).

According to the present findings, which are consistent with previous studies, although the central nervous system has limited capacity to regenerate damaged neurons, it has high capacity to reorganize its neural circuits in response to physical damage. This reorganization occurs in the remaining healthy neurons, where sprouting, supersensitivity, and changes in the number of receptors and receiving areas are observed. In addition, the nervous system has the ability to compensate for behavioral deficits by using neuronal circuits, which are not normally involved in the control of motor behaviors. However, the role of learning processes in compensating for behavioral defects should not be ignored (Schwarting et al. 1991).

The number of apomorphine-induced turnings within 30 min indicated a lesion in the dopaminergic system on the side of 6-OHDA injection. Previous studies show that apomorphine acts as a dopamine agonist and can be coupled with dopamine receptors. By developing an experimental model of PD using 6-OHDA, the process of dopamine release from the axonal terminals of nigrostriatal pathway is impaired. In this situation, the number of dopamine receptors in the striatum increases in a compensatory manner. Therefore, apomorphine, as a dopamine agonist, can bind to enhanced receptors, increase the response of the striatum on the lesion side, and improve the number of rotations on the side of the lesion (Cleren et al. 2008).

In unilateral animal models of PD, a rotation number increasing following the administration of dopamine agonists, such as apomorphine, is considered a valid measure for determining the reduction of dopamine in the target region of the nigrostriatal system. In the present study, apomorphine-induced rotational behaviors indicated a significant increase in the contralateral rotations of PD rats, compared to the control group. The results indicated that the number of neurons in the striatum and SN(pc) significantly reduced after the induction of PD, compared to the healthy control group. Moreover, the number of neurons in the striatum and SN(pc) increased significantly in the cell transplantation, melatonin, and cell transplantation + melatonin groups, compared to the PD group. Also, the number of neurons in the striatum and SN(pc) was higher in the cell transplantation + melatonin group, compared to the other two treatment groups.

6-OHDA, with toxic effects on the production of oxygen-derived free radicals, destroys dopaminergic neurons on the injection side of the midbrain and leads to dopamine depletion on the same side of the striatum. Generally, cell death occurs in two stages. Acute death starts 12 h after injection and lasts approximately 7–10 days after lesion development (Agrawal et al. 2004). Cell transplantation in the striatum maintains dopaminergic terminals in the striatum/SN and leads to the proliferation and neuralization of newborn cells in the subventricular zone, without affecting the number of glial cells. Our findings showed that cell transplantation could contribute to cell survival and lead to the specific development of immature cells towards neurons in animal models of PD (Cova et al. 2010).

Western blot analysis of caspase-3 in the groups showed that expression of this protein in the 6-OHDA group was higher than the control and treatment groups. Moreover, the results of GSH test showed an increase in the level of this protein in the treatment groups (cell transplantation, melatonin, and cell transplantation + melatonin), compared to the 6-OHDA group. Also, expression of this protein in the cell transplantation + melatonin group was higher than two other treatment groups.

The results of various studies show that 6-OHDA produces reactive oxygen species (ROS), which has an essential role in the death of dopaminergic neurons. ROS also causes phosphorylation of p38 and caspase-8 and results in cell death associated with caspase-3 in cultured mesencephalic cells (Weinreb et al. 2010). The apoptotic effect of 6-OHDA was first shown by Walkinshow and Waters in 1994. They show that low concentrations of 6-OHDA induced morphological and biochemical symptoms of apoptosis (Walkinshaw and Waters 1994). Moreover, 6-OHDA induces apoptosis in dopaminergic cells of human neuroblastoma. Therefore, these cells show morphologically and biochemically changes, such as cellular shrinkage, chromatin density, and DNA fragmentation.

Cytochrome C can activate caspases and induce apoptosis after release from mitochondria (Shieh et al. 1997). Oxidative stress has an essential role in the pathogenesis of PD and causes damage to intracellular lipids, proteins, and DNA, resulting in the degeneration and death of dopaminergic neurons (Lee and Liu 2008). Our study showed that melatonin exerts protective effects on intracellular ROS production induced by 6-OHDA. Excessive production of ROS can greatly disturb the mitochondrial membrane and cause the release of apoptosis-inducing agents and activation of caspase cascade (Lee and Liu 2008).

Disruption of mitochondrial function can activate cell death by releasing apoptosis-inducing agents, such as procaspases, caspase activators, and caspase-independent agents. The released cytochrome C can cause apoptosis by activating downstream caspases, such as caspase-3 (Wu and Bratton 2013). Moreover, TH-positive cells, secreting dopamine, extend their axons to the host striatum following cell transplantation. They create functional synaptic relationships, and improve symmetric movements (Tajiri et al. 2014).

Reduction of neuronal death in the cell transplantation groups can be directly related to the improvement of motor movement. The function of live neurons is facilitated through cell–cell interactions and secreted media from the cells, which contain multiple factors, such as chemokines, cytokines, trophic factors, and IncRNA as signaling molecules, as well as anti-inflammatory factors possibly involved in the epigenetic mechanisms of neuroprotection and synaptic plasticity (Tajiri et al. 2014).

Melatonin increases TH expression and reduces the effects of 6-OHDA. Generally, apoptosis is a cellular process with more than one pathway. The proapoptosis interaction of BAX and antiapoptotic interaction of BCL-2 determine the cell fate by regulating mitochondrial membrane and releasing cytochrome C (Yildirim et al. 2014). Moreover, caspase protease activation is required for mitochondrial related death, when releasing cytosol, cytochrome C, procaspase-9, and apoptosis protease–activating factor 1 produce apoptosomes and activates caspase-3; this finding is consistent with our results, which showed that caspase-3 increased in the 6-OHDA group. On the other hand, melatonin can decrease caspase-3 effects on signaling pathways by reduction of lipid peroxidation and altering the membrane transmission (Bonnefont-Rousselot and Collin 2010).

## Conclusion

In conclusion, combination therapy with cell transplantation and the use of antioxidants is a new method of treating various diseases. Given that, transplanted cells show limited survival at the transplant site because of some factors such as free radicals, reduction of nutritional factors and apoptosis. Given that human knowledge today seeks to replace damaged neurons in the nervous system in neurodegenerative diseases such as Parkinson's and also in an effort to preserve transplanted cells, the anti-apoptotic role of melatonin is involved in the preservation and survival of transplanted cells by inhibiting cytochrome C secretion as an anti-apoptotic factor. It seems that the co-administration of dopaminergic neurons and melatonin can decrease the free radicals and increase the survival of transplanted neurons and have therapeutic effects on motor disabilities in Parkinson's disease.

## Data Availability

Data will be available after request.
